# Cytokine modulation in abdominal septic shock *via* the crucial role of IL-6 signaling in endothelial dysfunction

**DOI:** 10.3389/fmed.2023.1042487

**Published:** 2023-03-01

**Authors:** Takuya Ueno, Toshiaki Ikeda, Masaaki Okihara, Isao Akashi, Takayoshi Yokoyama, Yu Kihara, Osamu Konno, Yuki Nakamura, Hitoshi Iwamoto, Yu Ueno, Anil Chandraker

**Affiliations:** ^1^Department of Kidney Transplantation Surgery, Tokyo Medical University Hachioji Medical Center, Tokyo, Japan; ^2^Transplantation Research Center, Renal Division, Brigham and Women's Hospital, Harvard Medical School, Boston, MA, United States; ^3^Division of Critical Care and Emergency Medicine, Tokyo Medical University Hachioji Medical Center, Tokyo, Japan

**Keywords:** inflammatory cytokines, Endothelial (dys)function, abdominal septic shock, Renal replacemant therapy, PMX (polymyxin B-immobilized fiber column)

## Abstract

**Background:**

Early recovery from shock improves prognosis in septic shock patients. We determined whether cytokine modulation by Continuous Renal Replacement Therapy (CRRT) following acute care surgery resulted in stable hemodynamics in them. To investigate our hypothesis, we measured proinflammatory cytokines IL-6, IL-1ra and the coagulation cascade activator plasminogen activator inhibitor-1 (PAI-1) following CRRT with polymyxin B immobilized fiber (PMX-DHP) which has been utilized as an adjuvant treatment option for patients with severe septic shock.

**Methods:**

66 septic shock patients requiring 2 h direct hemoperfusion therapy PMX-DHP were included. 36 patients of them also received continuous hemodiafiltration (CHDF) after performing PMX-DHP. Circulatory dynamics and levels of inflammatory mediators, namely IL-6, IL-1ra, and PAI-1 were assessed before, immediately after, and 24 h initiation of PMX-DHP.

**Results:**

Mean Arterial Pressure (MAP) rose intentionally by PMX-DHP just after enforcement 24 h later (*p* < 0.01). Levels of IL-6, IL-1ra, and PAI-1 significantly decreased after PMX-DHP (*p* < 0.05) and this trend was observed up to 24 h post initiation of PMX-DHP (*p* < 0.05). IL-6 modulation by PMX-DHP was enhanced with using CHDF and there was a significant correlation between IL-6 and MAP (*p* < 0.0001). In addition, levels of Il-6 and PAI-1 showed a significant correlation.

**Conclusion:**

Our data showed employing CRRT as cytokine modulators could be an additional therapeutic strategy to improve septic shock outcomes *via* the crucial role of IL-6 signaling in endothelial dysfunction.

## Introduction

Severe sepsis places a large burden on health care systems, with a short-term mortality of 20–25%, reaching up to 50% when shock is present (1). Therefore, there is an urgent unmet clinical need to develop novel therapies to improve patient outcomes and survival rate. *In 2007,* the first systematic review of PMX-DHP (Toray Industries Inc., Tokyo, Japan) showed that PMX-DHP lowers mortality from sepsis, reduces the need for dopamine, and increases MAP and PaO_2_/FiO_2_ ratio (2) and Vincent et al. also reported its beneficial effect on cardiac and renal function in severe sepsis or septic shock (3). In Japan, hemoperfusion using PMX-DHP is utilized as an adjuvant treatment option for patients with severe septic shock, with the aim of reversing the shock state and stabilizing hemodynamics. We reported levels of inflammatory mediators such as endotoxin, TNFα, IL-1, and IL-6 are decreased after PMX-DHP (4–6). It has been postulated that the benefits of treatment with PMX-DHP in septic states may be mediated through modulation of levels of these and other pro-inflammatory cytokines. Moreover, the effectiveness of early use of PMX-DHP in abdominal septic shock showed that PMX-DHP added to conventional therapy led to improved hemodynamics and organ dysfunction and significantly reduced 28-day mortality (7). We also have an access to perform another Continuous Renal Replacement Therapy (CRRT) named CHDF using a polymethyl methacrylate (PMMA) membrane hemofilter (PMMA-CHDF; CHDF) in septic shock. As data from Drs. Hirasawa and Oda showed cytokine removal using CHDF to be effective for the treatment of severe sepsis and septic shock (8–11), CHDF acts as a cytokine modulatory therapy in the treatment of patients with severe sepsis/septic shock and resulted in earlier recovery from severe sepsis (11). No data are yet available on the importance of CRRT in regulating hemodynamics through cytokine modulation in septic shock patients following acute care surgery. In addition, inflammatory cytokine does not seem to have a dominant effect on systolic or diastolic myocardial dysfunction in real-life sepsis (12).

We hypothesized cytokine modulation by CRRT resulted in stable hemodynamics in abdominal septic patients following acute care surgery. To assess this, we measured levels of inflammatory cytokines at three different time points during treatment and examined hemodynamic stability in each treatment arm. These cytokines were described in former reports where we measured them as useful biomarkers that predict patients’ prognosis in sepsis (4–6, 13).

## Materials and methods

### Study design

Single-center prospective observational study. All acute abdomen cases treated at Tokyo Medical University Hachioji Medical Center from 2012 to 2017.

### Setting

Intensive care unit (ICU) and emergency department (ED) at a university hospital.

### Human subjects protection

Informed consent was obtained from all participating subjects or their families (written). This study was approved by the Institutional Review Board of Tokyo Medical University.

### Patients

We enrolled 66 patients who fulfilled the criteria of septic shock (14) requiring 2 h PMX-DHP to maintain adequate hemodynamics despite administration of early goal-directed therapy, vasopressors, and acute care surgery including source control (Table 1).

**Table 1:** Demographic characteristics and underlying diseases of patients.

	PMX-DHP (Group A)	PMX-DHP + CHDF (Group B)
Characteristic	**(**Mean and standard error)	
Patients (Survivor/Non-survivor) (*n*)	30 (21/9)	36 (22/14)
Age (years)	68.6 ± 10.6	69.5 ± 10.1(ns = 0.97)
Female sex-no (S/NS)	9 (5/4)	9(5/4) (ns)
SIRS	3.0 ± 0.7	3.7 ± 0.5 (ns = 0.051)
APACHE II Score	19.19 ± 6.5	27.9 ± 6.9***
SOFA score	8.0 ± 2.9	11.3 ± 2.9(ns = 0.059)
IL-6	26,353 ± 48,400	49,656 ± 79,345 (ns = 0.094)
Underlying disease(*n*)		
Peritonitis	24	24
Strangulated ileus	5	5
Biliary tract infection	1	6
Abscess (Kidney, liver)	0	1

Sixty-six patients were divided into 2 groups, where all patients received 2 h PMX-DHP; either alone (Group A) or with addition of CHDF (Group B) for the following 24 h. Baseline APACHE II score was significantly different between patients in Group A and B. (***p < 0.001), which indicated that Group B patients are under severe condition compared to Group A patients.

### Resuscitation strategy

First we gave a 500-mL bolus of crystalloid every 30 min to achieve a central venous pressure of 8 to 12 mmHg. If the mean arterial pressure was less than 65 mmHg, vasopressors were given to maintain a mean arterial pressure of at least 65 mmHg. If the mean arterial pressure was greater than 90 mmHg, vasodilators were given until it was 90 mmHg or below. Since we corrected data up to 2017, some patients received an EGDT (15) strategy at earlier timepoints before employing 2 h therapy with PMX-DHP, but the other did not because we followed the guideline published in 2017 (16, 17). Although the current SSCG (18, 19) also does not recommend the fluid resuscitation like the former one based on three subsequent large multicenter RCTs (20–22), our center has been basically using crystalloid fluid therapy as an initial resuscitation to improve patients’ unstable hemodynamics dysfunction for years.

Dialysis settings: PMX-DHP/CHDF were performed using a double-lumen catheter placed in the femoral vein, subclavian vein, or internal jugular vein, at a blood flow rate of 80 mL/min, using nafamostat mesilate mesylate (Torii Co., Ltd., Tokyo, Japan) as an anti-coagulant (5, 6, 13, 23). We measured activated clotting time (ACT) on a regular basis before each session.

### Study protocol

**Figure 1:**
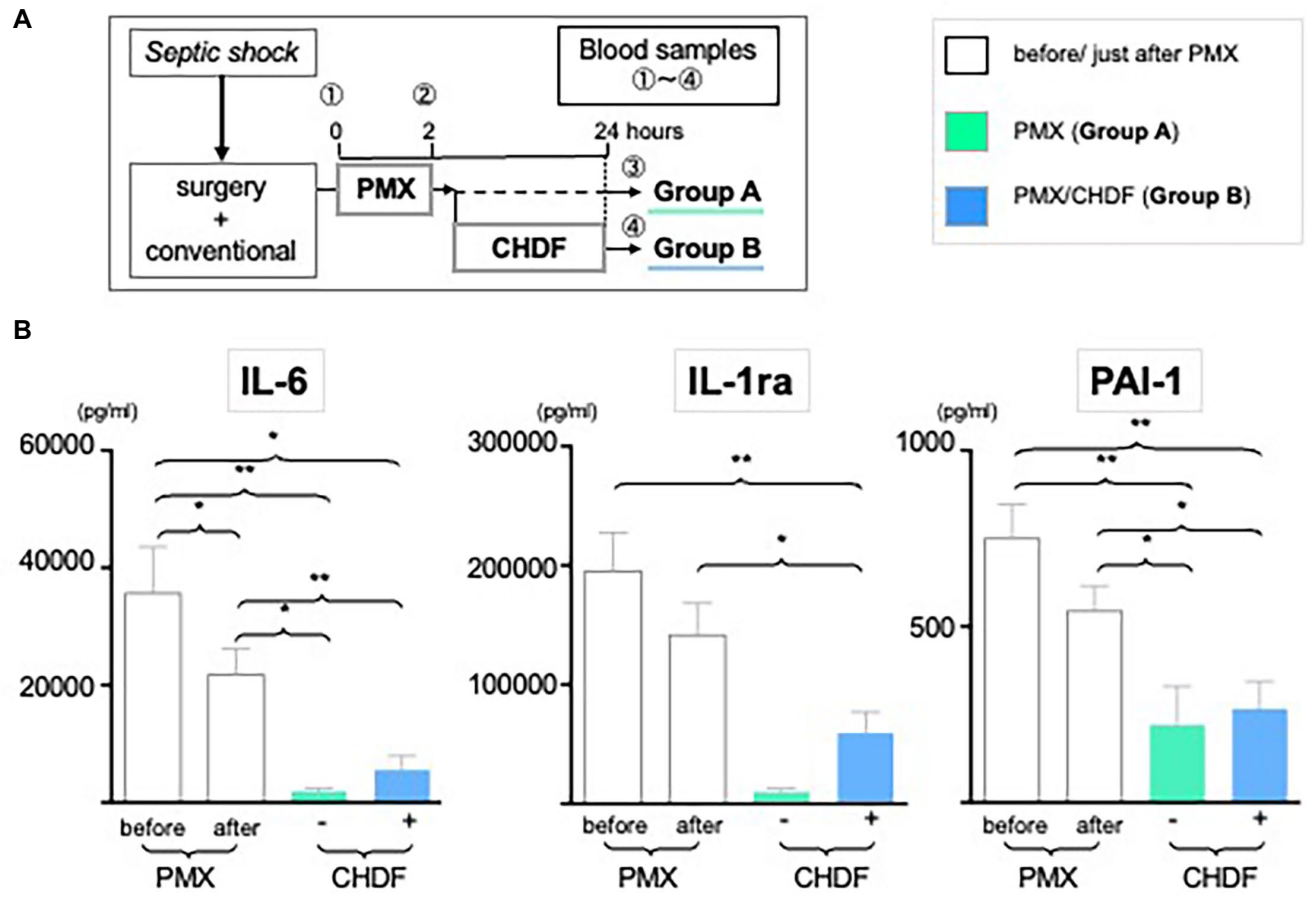
Dynamic level of IL-6, PAI-1 and IL-1ra following CRRT. **(A)** Study design: all enrolled patients received a conventional therapy with PMX-DHP and then they were divided into two groups with (Group B, blue) or without (Group A, green) CHDF right after PMX-DHP. We measured various cytokines levels before, just after, and 24 h initiation of PMX-DHP. **(B)** All 3 panels showed significant reduction of IL-6, IL-1ra, and PA-1 in 2 different time points (just after, 24 h later) compared to before PMX-DHP. This trend is more remarkable in Group A (without CHDF) at 24 hours; possibly reflecting the greater disease severity in patients enrolled in Group B. (*p < 0.05, **p < 0.01).

All patients received fluid therapy, antibiotic therapy, and 2 h therapy with PMX-DHP. 30 patients received only PMX-DHP with conventional treatment (Group A) and 36 patients received both PMX-DHP and CHDF in addition to conventional treatment (Group B) (Figure 1A). The decision to add CHDF was basically based on high APACHE II score (>20) of patients. We measured IL-6, IL-1ra, and PAI-1 at three different time points: (1) before, (2) just after, and (3) 24 h post initiation of PMX-DHP (Figure 1A).

### Measurements

**Figure 2:**
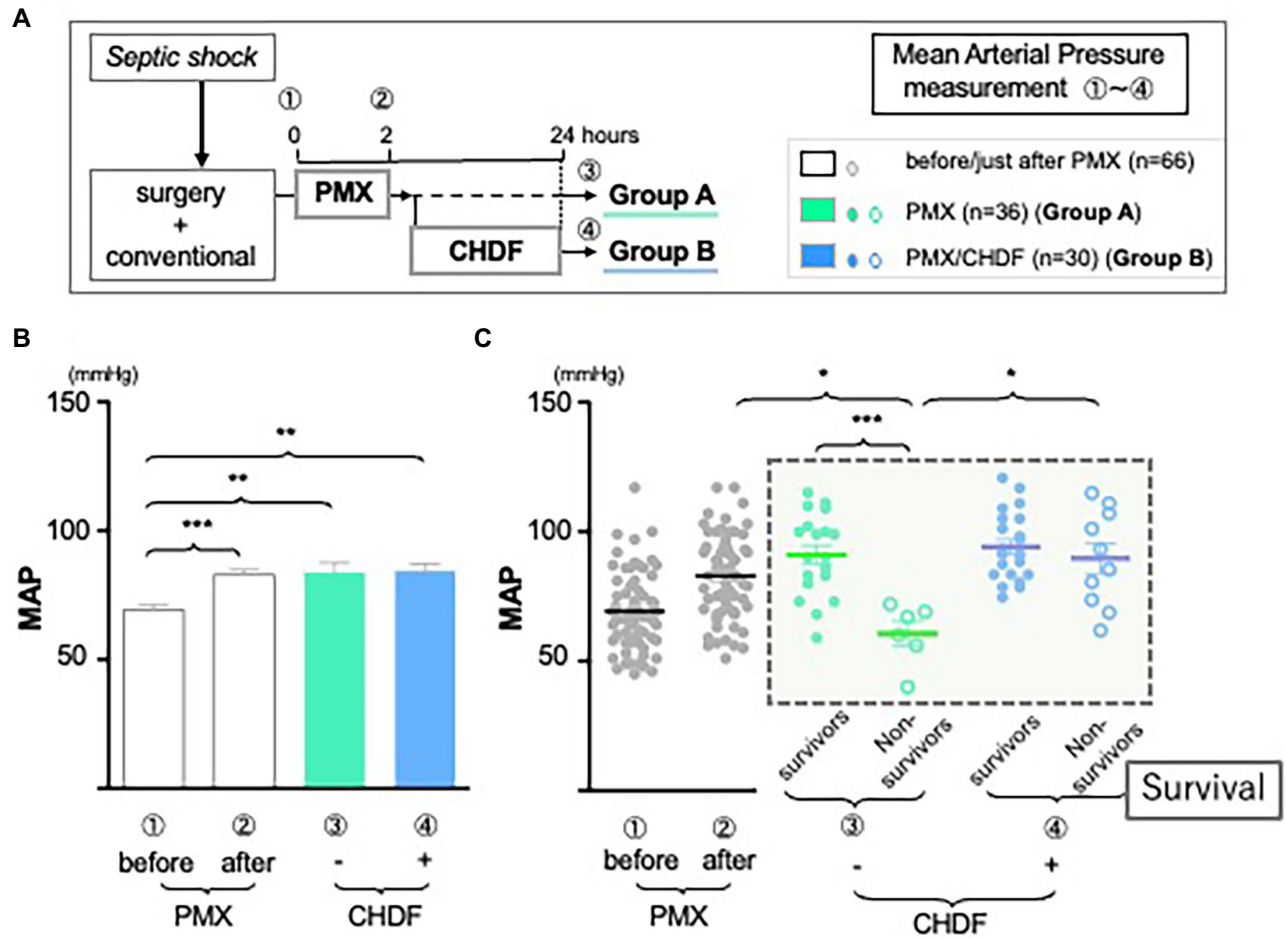
The role of CRRT in hemodynamics. **(A)** *Study design*: all enrolled patients received a conventional therapy with PMX-DHP and then they were divided into two groups with (Group B, blue) or without (Group A, green) CHDF right after PMX-DHP. We measured MAP before, just after, and 24 h initiation of PMX-DHP. **(B**,**C)** Changes in mean arterial pressure (MAP) in response to PMX-DHP and CHDF: Significant elevation in MAP was observed up to 24 h both groups after treatment with PMX-DHP **(B)**. We then subdivided those groups by survivors and non-survivors. MAP of non-surviving patients in Group A was significantly lower compared to survivors **(C)**. The same effect was not observed in patients in Group B (*p < 0.05, **p < 0.01, ***p < 0.001).

Blood samples were collected from an arterial line and Enzyme-linked immunosorbent assay (ELISA) and enzyme immunoassay (EIA) methods were used for the measurement of IL-6, IL-1ra, and PAI-1, respectively as we described before (24). We then evaluated the effect over time of PMX-DHP and CHDF on those levels in patients with abdominal septic shock. Mean arterial pressure was recorded as part of routine clinical care and compared between groups at those three different time points following PMX-DHP (Figure 2A).

### Statistical analysis

For statistical analysis, the GraphPad Prism software program (version 5; GraphPad Prism, La Jolla, CA) was used. Mean and standard error of mean (SEM) were calculated for each group. Comparison between groups was performed by a Student’s *t*-test. For multi-group comparisons of repeating data (variations over time) in the parametric data, a repeated measure analysis of variance (ANOVA) was used. If there were any significant differences in the repeated measure ANOVA, a one-way ANOVA with Bonferroni correction as a post-hoc.

analysis was used for multi-group comparisons of each single factor. The correlation coefficient was obtained using Pearson’s equation. Differences were considered to be significant at *p* < 0.05.

## Results

### Baseline and outcome characteristics of study patients

Demographic and baseline characteristics of enrolled patients, origins of septic shock and mortality rates are as follows (Age, systemic inflammatory response syndrome: SIRS, APACHE II, Survivor/Non-Survivor): Group A (68.6 ± 10.6, 3.0 ± 0.7, 19.9 ± 6.5, 21/9), Group B (69.5 ± 10.1, 3.7 ± 0.5, 27.9 ± 6.9*, 22/14) (*: *p* < 0.05). All patients received acute care surgery of peritonitis, strangulated ileus, biliary tract infection, and abscess (Table 1). Antimicrobial therapy was judged to be adequate when the patient received drugs to which each isolated microorganism was sensitive. The most commonly isolated microorganisms were Gram-negative bacteria, of which 10 strains were detected among the 66 patients. Of patients with peritonitis, strangulated ileus or biliary sepsis, Gram-negative bacteria were detected in 49 and Gram-positive bacteria were detected in 33 patients (Supplementary Table 1).

### Cytokines modulation by CRRT in abdominal septic shock

We measured serum levels of IL-6, IL-1ra and PAI-1 before (baseline), immediately after (2 h), and 24 h after initiation of PMX-DHP. IL-6 level was significantly decreased immediately after PMX-DHP (21807vs. 35,683 pg/mL, *p* < 0.05 as compared to baseline) with a further decrease seen by 24 h (1888 pg/mL, *p* < 0.05 as compared to baseline). The addition of CHDF did not further impact on IL-6 levels. Similarly, IL-1ra level was significantly decreased at 2 h (156,325 pg/mL, *p* < 0.01 as compared to baseline) with a further fall by 24 h after PMX-DHP in both groups (47,331 pg/mL, *p* < 0.001 as compared baseline) (Figure 1B). Immediately after PMX-DHP, PAI-1 level also significantly reduced from pre-treatment baseline levels (543.8 vs. 749.9 pg./mL, *p* < 0.05). This finding was sustained at 24 h (219 pg/mL, *p* < 0.01 as compared to baseline) in both groups. Similar to other cytokines assayed, the addition of CHDF did not further augment this fall in PAI-1 (Figure 1B). Across all three cytokines, a small, non-significant increase was seen in patients treated with CHDF vs. PMX-DHP alone.

### CRRT affected and significantly improved hemodynamics

As we reported in a systematic review of PMX-DHP published in *Crit Care* (2), the current study also showed mean arterial pressure (MAP) significantly improved and was sustained up to 24 h after treatment with PMX-DHP (Group A: MAP pre vs. immediately post PMX-DHP: 71.0 mmHg vs. 85.4 mmHg *p* < 0.001; vs. 83.8 mmHg at 24 h, *p* < 0.01; Group B: MAP pre vs. immediately post PMX-DHP: 68.0 mmHg vs. 80.8 mmHg, *p* < 0.001; vs. 83.0 mmHg at 24 h, *p* < 0.001). There was no detectable difference between MAP immediately after PMX-DHP and 24 h later in either group (Figure 2B). However, MAP was maintained at this higher level after CHDF in both survivors and non-survivors (Figure 2C, Group B as blue dots). In contrast, after PMX alone, a significant fall in MAP was observed in the subgroup of patients who did not survive (Figure 2C, Group A non-survivor as green circled, *p* < 0.001 compared to survivors).

### Significant decrease of IL-6 levels was not observed in non-survivors compared to survivors

**Figure 3:**
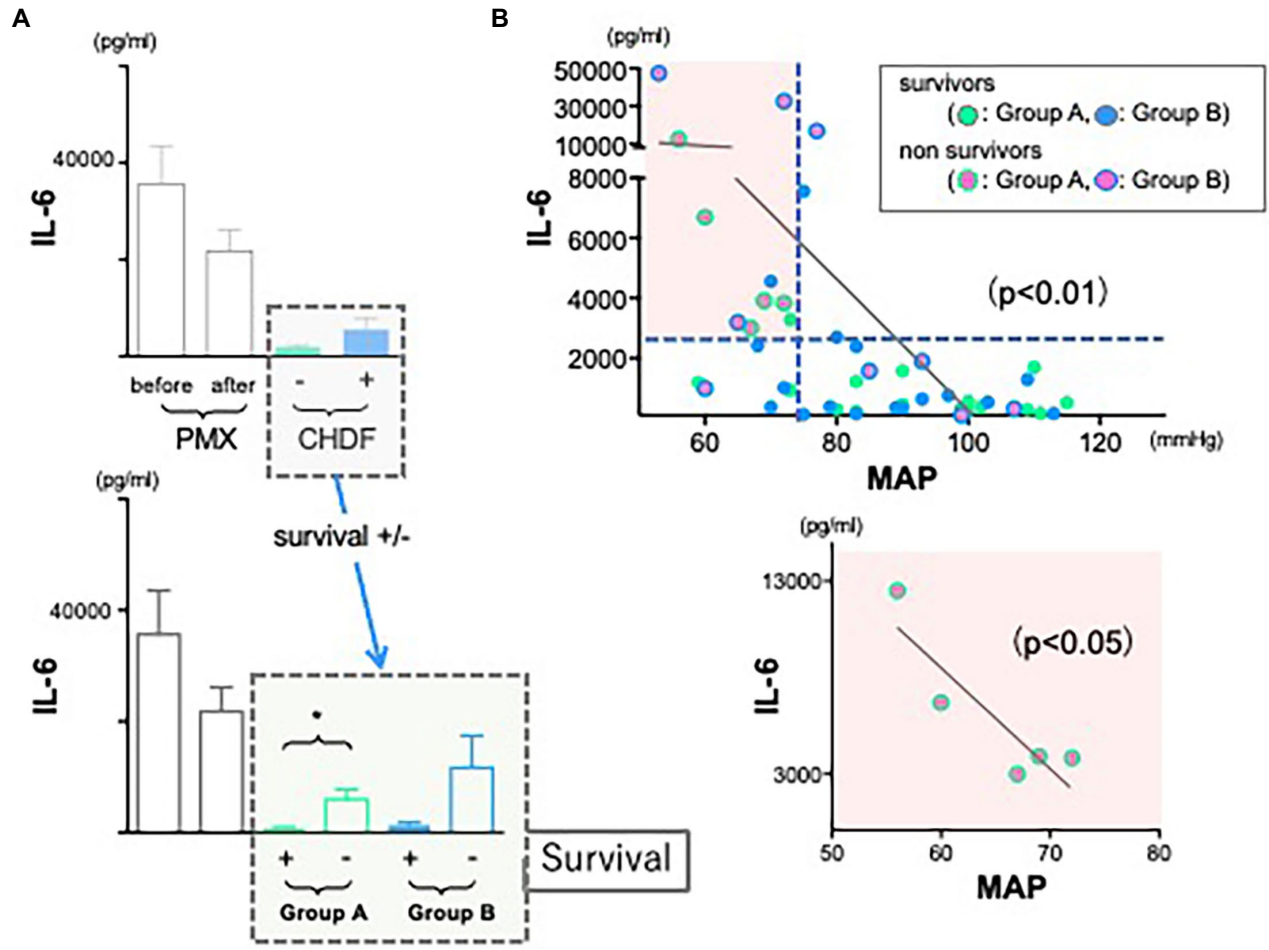
The role of IL-6 in hemodynamics and outcomes. **(A)** Although the use of CRRTs resulted in the reduction of IL-6 significantly in both groups, levels of IL-6 in both non-survivors were much higher than those of survivors (*p < 0.05). **(B)** There is a significant negative correlation between IL-6 and mean arterial pressure (*r* = 0.190, *n* = 45, *p* < 0.001). The correlation coefficient was obtained using Pearson’s equation.

We hypothesize that this may reflect differences in underlying disease severity between groups. Therefore, we divided all patients data into 4 different groups (not only +/− CHDF but also survivors/non-survivors) and measured cytokine levels, respectively. Employing CRRT resulted in cytokines reduction of IL-1ra and PAI-1 in both survivors and non-survivors similarly (Group A and B) (data not shown). However, there was a significant difference between survivors and non-survivors of Group A in cytokine reduction of IL-6 (*p* < 0.05). Survivors in Group A showed significant reduction of IL-6, however, its level of non-survivors (Group A) showed significantly higher compared to that of survivor (Figure 3A, green bars). This trend was also observed in non-survivors of Group B.

### Correlation between IL-6 and mean arterial pressure

As we tried to investigate to find some connections between levels of inflammatory cytokines and cardiac dysfunction in sepsis, we expected that cytokine modulation by CRRT resulted in stable hemodynamics in abdominal septic patients following acute care surgery. Therefore, we returned to the observation that we found (Figure 2C, black dotted square field). In contrast to non-survivors in Group A (green circled dots), MAP was significantly and well maintained in non-survivors of Group B (blue circled dots, *p* < 0.05). We investigated whether IL-6 level may affect hemodynamics. We found there was a significant negative correlation between IL-6 and MAP (Figure 3B, upper panel) (*p* < 0.001). Above all, there was a significant negative correlation in non-survivors of Group A (lower panel).

### Increased IL-6 cytokine could be a trigger to affect an increase of PAI-1 levels in hyperdynamic state

**Figure 4:**
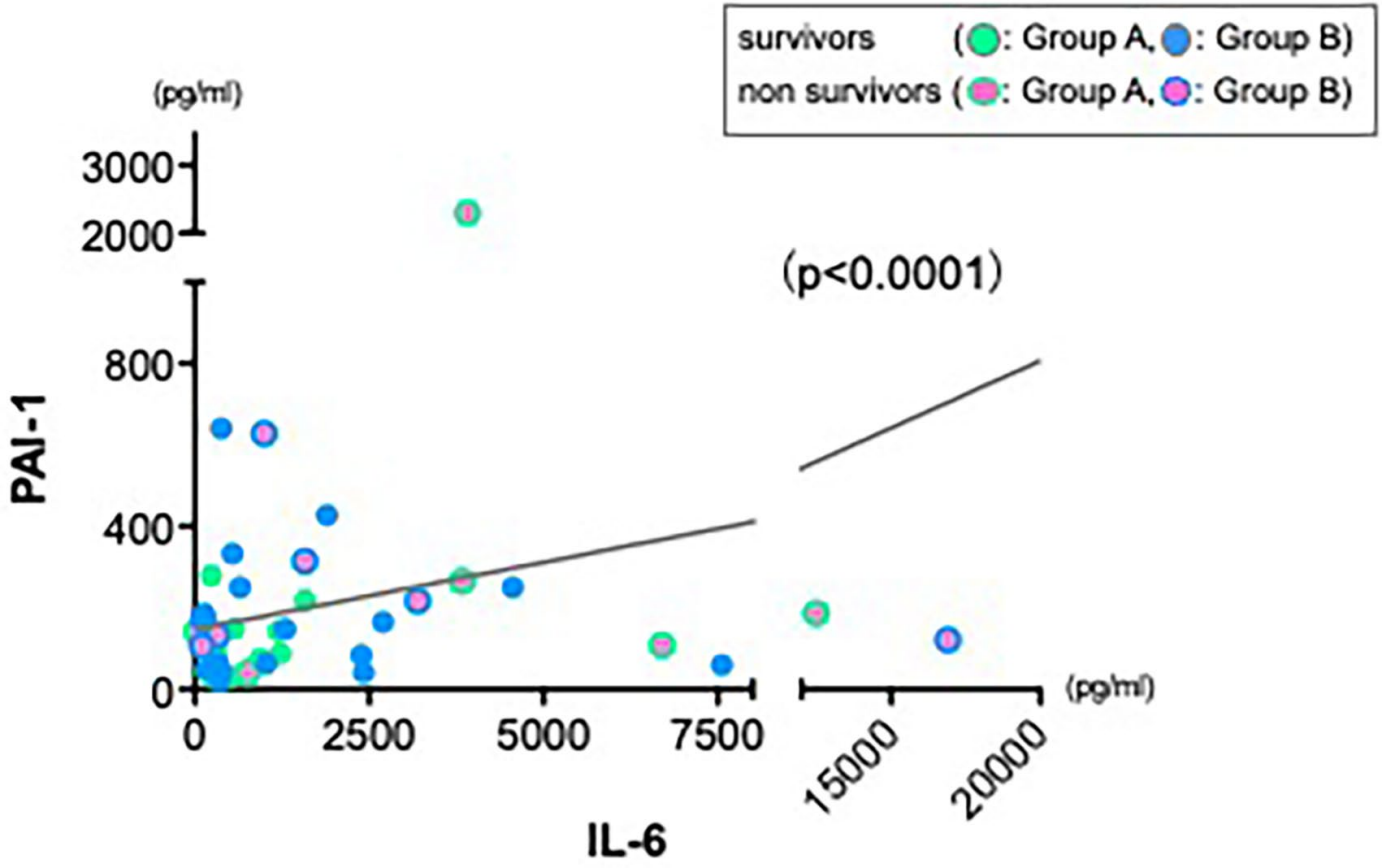
Strong positive correlation between IL-6 and PAI-1. Data from 24 h showed a significant positive correlation between IL-6 and PAI-1 (*r* = 0.325, *n* = 45, *p* < 0.0001).

As various inflammatory cytokines are affecting each other in cytokine release syndrome (25), we hypothesized whether increase of IL-6 cytokine could be a main trigger to the severe hyperdynamic state in sepsis or that of other cytokines could. Therefore, we also returned to the first observation showed in Figure 1. In contrast to other cytokines reductions, levels of PAI-1 were not reduced sufficiently at 24 h in both groups. First, we also investigated a correlation between PAI-1 and MAP as we performed and showed in Figure 3. There was no correlation between them (data not shown). Therefore, we next investigated a relation between IL-6 and PAI-1 at 24 h to find an indirect role of PAI-1 in hemodynamic profile of severe sepsis and septic shock. There was a significant positive correlation between IL-6 and PAI-1 (Figure 4) (*p* < 0.0001).

## Discussion

The basic principles of operative treatment of intraabdominal sepsis are source control to eradicate the underlying lesion, peritoneal toilet to evacuate bacterial inocula and pus, and operative intervention to treat persistent or recurrent infections (26). Despite remarkable developments in critical care medicine and extensive study of patients with tertiary peritonitis, the associated mortality rate remains nearly 30% (27). Moreover, Annane et al. reported a short-term mortality reaches up to 50% when shock is present (1).

In this study, we enrolled 66 patients who presented with intra-abdominal sepsis, shock and increased IL-6 levels. All received acute care surgery and standard goal-directed therapy, as per SSCG, with the addition of hemoperfusion therapy. In our entire cohort, 23 patients (34.8%) died, while 43 of 66 (65.2%) patients survived. Consistent with our previously published papers (2, 5, 23, 24); significant increases in MAP were seen after treatment with PMX-DHP. This beneficial effect was observed up to 24 h in both groups. In addition, Figure 2C showed marked difference in MAP at 24 h between survivors and non-survivors in Group A, indicating that a fall in MAP between completion of PMX-DHP and 24 h later may be a negative prognostic indicator. Furthermore, in Group B, MAP was better maintained in both survivors and non-survivors, suggesting that CHDF could prolong beneficial effects of PMX-DHP on hemodynamics, even in a sicker cohort of patients. In summary, these data may suggest that applying PMX-DHP plus CHDF treatment in the first 24 h could potentially improve the clinical outcome of abdominal septic shock through optimized hemodynamics.

With this study, we further sought to investigate a novel therapeutic application of PMX-DHP and CHDF; acting as cytokine modulatory therapies. We found significant reduction of IL-6, IL-1ra, and PAI-1 levels with both of these treatments (Figure 1; Supplementary Table 3). These proinflammatory cytokines (28, 29) have previously been proposed as potential therapeutic targets in sepsis. In particular, IL-6 modulation by PMX-DHP can be enhanced with using CHDF (Figure 1B), which may suggest that the current combination treatment can maintain not only early stage of sepsis but also late phase of sepsis *via* some cytokine cascade. We also need to shed a light on anticoagulant agent where we employed the renal settings during the ICU days. As we have been using specific anticoagulant agent named nafamostat mesylate described in above during the dialysis sessions, employing this agent showed also beneficial effects in the reduction of increased inflammatory cytokines and mediators such as IL-6, TNF-a and PAI-1 in sepsis and other critical illness (30–32). In fact, 25–50% of septic patients have a risk to be more severe clinical status such as DIC (33), therefore, we need to pay more attention to choose the agent once when data from patients starts to show a tendency with coagulation dysfunction.

Moreover, septic patients with low MAP plus elevated or unchanged IL-6 level following PMX-DHP could be a high-risk group in their prognosis (Figure 3B, red square field). As there was a negative significant correlation between IL-6 and MAP in non-survivors of Group A (Figure 3B, lower panel), maintaining cardiac function *via* cytokine modulation following CHDF could be an alternative artificial life support in hemodynamic profile of severe sepsis and septic shock.

**Figure 5:**
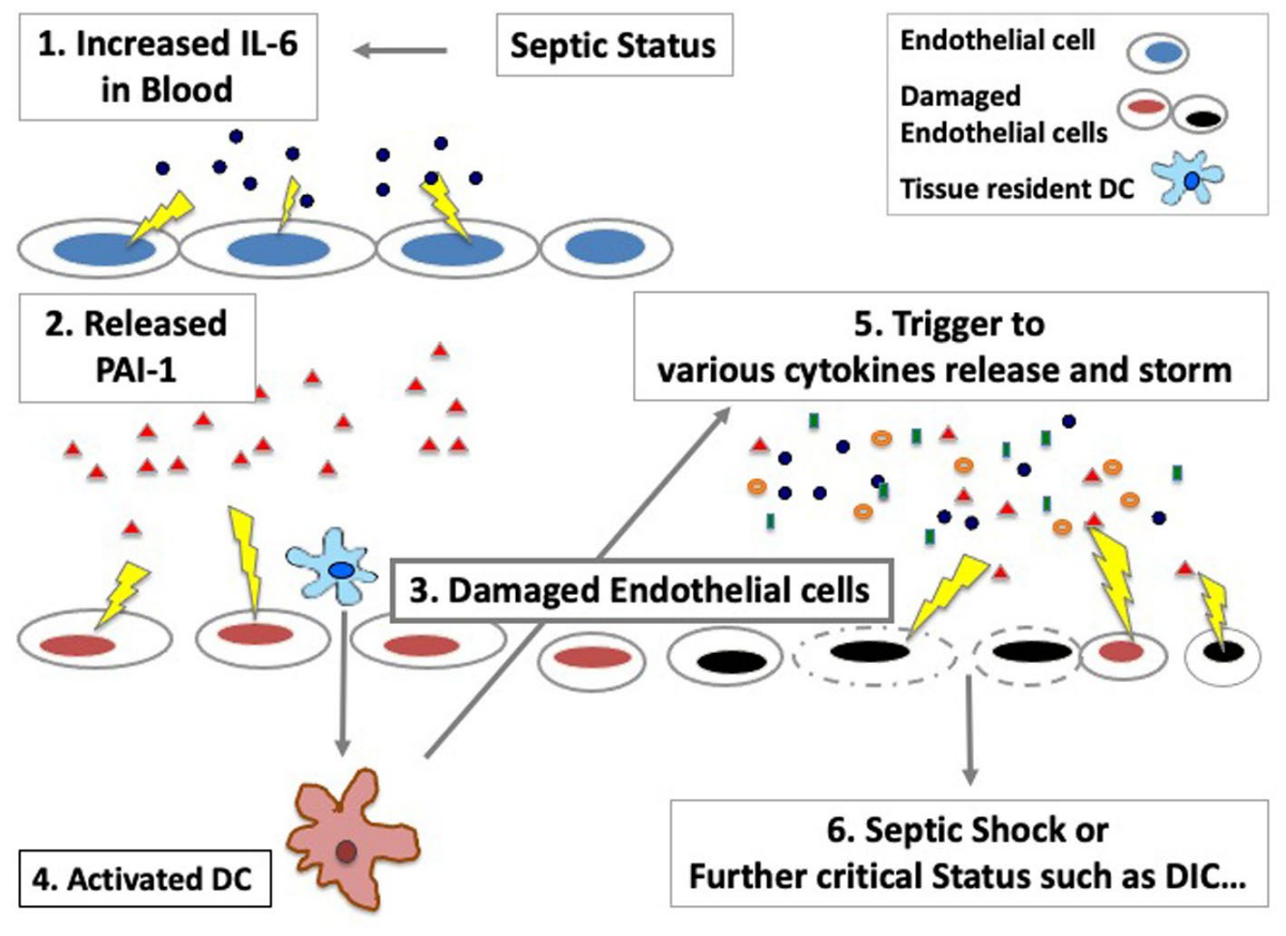
The important roles of endothelial cells in sepsis in the maintenance of cytokine storm. Various cytokines can be released *via* activated/damaged innate immune cells, which would affect not only hemodynamics dysfunction but also an increase of the risk of coagulation system disorders such as DIC (Disseminated Intravascular Coagulation).

Since the pathophysiology of sepsis is still poorly understood, we raised a new therapeutic approach with using CRRT in sepsis where we reported the important roles of innate immune cells such as dendritic cells and endothelial cells in the maintenance of homeostasis in patients with sepsis and transplantation (6, 34, 35). As we showed in Figure 5, increased IL-6 cytokine could be a main trigger to the next phase of cardiac dysfunction *via* increase of PAI-1, which may lead to the further critical phase of sepsis.

Although we saw favorable effects of PMX-DHP and CHDF on hemodynamics and cytokine modulation, the present study has several important limitations. As a small, single center, retrospective analysis, it has inadequate power to draw conclusions regarding patient outcomes. Furthermore, due to its non-randomized design, where patients were assigned to receive CHDF based on high APACHE II score, and we cannot control for the effect of confounding by indication. Although there were no significant differences in the level of IL-6 between Group A and B, APACHE II scores were significantly different (Table 1). This may indicate other, unmeasured factors that could also impact on the cytokine levels and affect response to therapy. Although we saw significant differences in MAP between groups, additional data such as lactate level and SvO2 would also have been helpful to more fully assess end-organ perfusion. However, such data was not available. To date, PMX-DHP has been used in more than 100,000 patients with a very low incidence of adverse events and high tolerability, which resulted in 120 English language publications reporting on over 2000 patients. Only several randomized controlled trials including ongoing studies such as European pilot trial, EUPHAS (7), ABDO-Mix (36), and EUPHRATES (37) have been performed and some systematic reviews (38–40) and cohort studies (41, 42) in the field were reported (Supplementary Table 2). Therefore, we do hope that the current ongoing study named TIGRIS which is carrying out at multicenter in the US, which would lead to prove the evidence (43).

We could enroll 66 septic patients in the current study, however, these were critically ill patients, treated with maximal available therapy and therefore the current study lacks a control group (conventional only) and CHDF only group. This limits our ability to truly quantify the cytokine modulating-effect of these therapies independently. Finally, we found a significant negative correlation between IL-6 and mean arterial pressure (Figure 3B) and we also found a strong positive correlation between IL-6 and PAI-1 (Figure 4), which suggests an important role of various cytokines modulation in the maintenance of endothelial cells viability *via* using CRRT.

## Conclusion

Drugs that specifically inhibit or neutralize endogenous inflammatory cytokines are in widespread use. The present study showed there are several beneficial effects of PMX-DHP plus CHDF treatment to promote optimized hemodynamics and modulate cytokine levels in ICU patients with severe sepsis following acute care surgery. This is the first report showing these advantages in abdominal septic shock patients and we showed an important role of endothelial cells in the maintenance of cytokine storm in sepsis.

## Data availability statement

The original contributions presented in the study are included in the article/Supplementary material, further inquiries can be directed to the corresponding author.

## Ethics statement

The studies involving human participants were reviewed and approved by Tokyo Medical University. The patients/participants provided their written informed consent to participate in this study.

## Author contributions

TU, TI, MO, IA, TY, YK, OK, YN, and HI participated in the performance of the research, performed the data collection, performed the statistical analysis, and contributed to the writing of the manuscript. TI and YU participated in the writing of the manuscript and performed review. TI and AC helped in the design of the study. All authors contributed to the article and approved the submitted version.

## Funding

The current data has been presented at the 2nd E-ISFA Congress in Vienna 2018 and supported by JSPS KAKENHI. TU is a recipient of Grant-in-Aid for Scientific Research (B: 17H04333) and (C: 20K08994).

## Conflict of interest

The authors declare that the research was conducted in the absence of any commercial or financial relationships that could be construed as a potential conflict of interest.

## Publisher’s note

All claims expressed in this article are solely those of the authors and do not necessarily represent those of their affiliated organizations, or those of the publisher, the editors and the reviewers. Any product that may be evaluated in this article, or claim that may be made by its manufacturer, is not guaranteed or endorsed by the publisher.

## Supplementary material

SUPPLEMENTARY TABLE 1 Characters of microorganisms.

SUPPLEMENTARY TABLE 2 Clinical outcome in the use of PMX-DHP. Over the last decades, roles of CRRT in septic field developed numeours findings and advantages to have a better outcomes.

SUPPLEMENTARY TABLE 3 Changes of Procalcitonin and HMGB-1 levels. Initial data in both markers are significantly increased in Group B and both levels were improved after the 2 h PMX-DHP in both groups compared to before the treatment (data not shown).
