# Supplementary material for: Cytokine modulation in abdominal septic shock via the crucial role of IL-6 signaling in endothelial dysfunction
Source: Front Med (Lausanne). 2023 Mar 1;10:1042487. doi: 10.3389/fmed.2023.1042487 (PMC10052569; doi:10.3389/fmed.2023.1042487)
Supplement: SUPPLEMENTARY TABLE 1 — Characters of microorganisms. [file Table_1.pdf]

| **Characteristic** | ***PMX-DHP*** | ***PMX-DHP* + *CHDF*** |
| --- | --- | --- |
| **Etiology of infection** | (n) | |
| ***Gram-negative bacteria*** | | |
| *Escherichia coli* | 10 | 14 |
| *Pseudomonas aeruginosa* | 5 | 10 |
| *Klebsiella pneumoniae* | 2 | 5 |
| *Enterobacter* | 0 | 2 |
| *Proteus mirabillis* | 1 | 0 |
| ***Gram-positive bacteria*** | | |
| *$\alpha$-Streptococcus* | 6 | 12 |
| *Methycillin-Resistant Staphylococcus Aureus* | 4 | 8 |
| *Staphylococcus aureus* | 0 | 2 |
| *Enterococcus sp* | 0 | 1 |
| ***Fungi*** | | |
| *Candida albicans* | 2 | 2 |
