# Supplementary material for: Cytokine modulation in abdominal septic shock via the crucial role of IL-6 signaling in endothelial dysfunction
Source: Front Med (Lausanne). 2023 Mar 1;10:1042487. doi: 10.3389/fmed.2023.1042487 (PMC10052569; doi:10.3389/fmed.2023.1042487)
Supplement: SUPPLEMENTARY TABLE 2 — Clinical outcome in the use of PMX-DHP. Over the last decades, roles of CRRT in septic field developed numeours findings and advantages to have a better outcomes. [file Table_2.pdf]

	PMX-DHP (Group A)	*PMX-DHP + CHDF (Group B)*
Characteristic	(Mean and standard error)	
Procalcitonin	35.64±41.5	18.9±61
HMGB-1	7.3±7.9	12.8±8.4
