# Supplementary material for: Cytokine modulation in abdominal septic shock via the crucial role of IL-6 signaling in endothelial dysfunction
Source: Front Med (Lausanne). 2023 Mar 1;10:1042487. doi: 10.3389/fmed.2023.1042487 (PMC10052569; doi:10.3389/fmed.2023.1042487)
Supplement: Supplementary file 4 [file Presentation_1.pptx]

## Slide 1
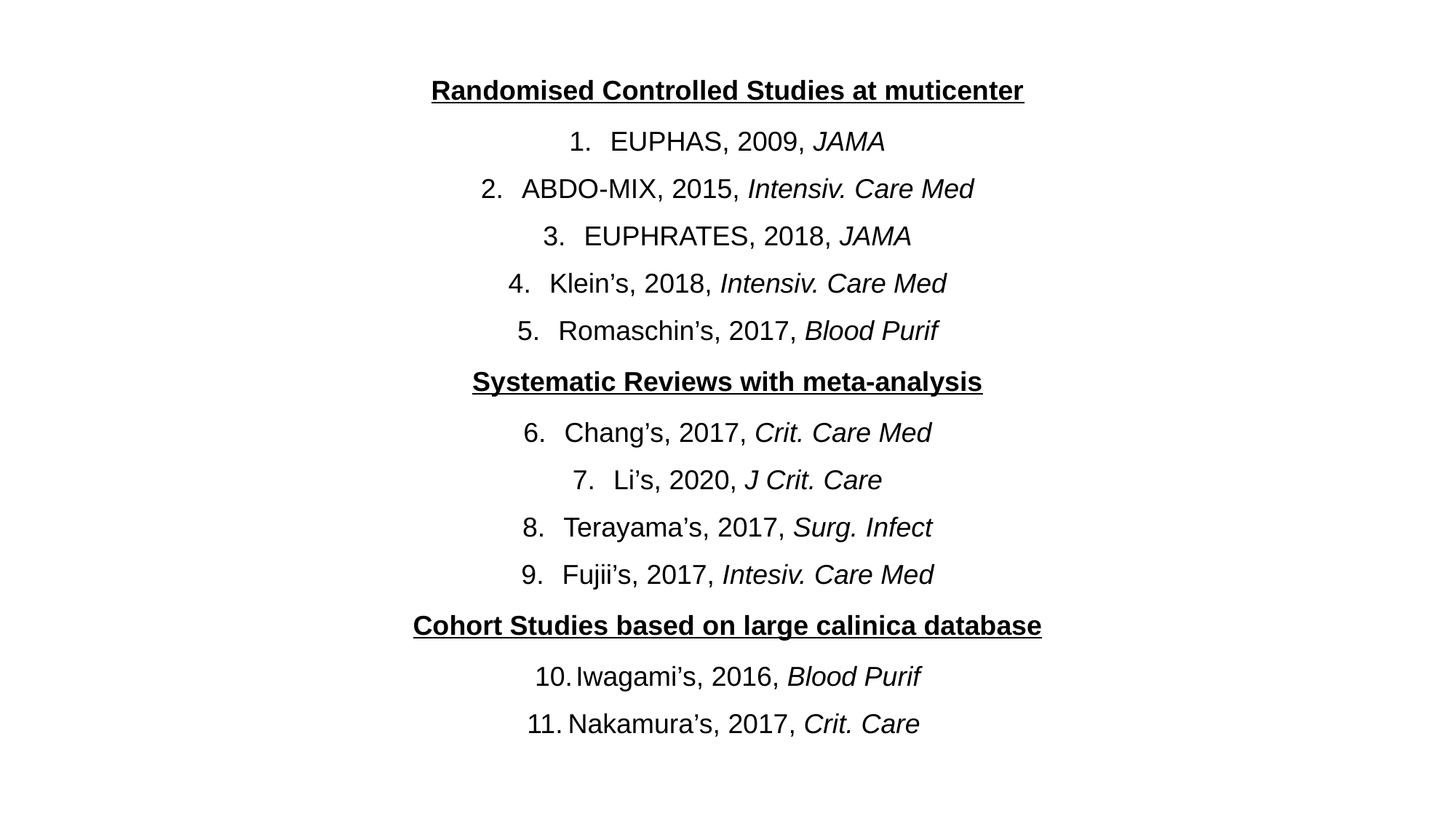

Randomised Controlled Studies at muticenter
EUPHAS, 2009, JAMA
ABDO-MIX, 2015, Intensiv. Care Med
EUPHRATES, 2018, JAMA
Klein’s, 2018, Intensiv. Care Med
Romaschin’s, 2017, Blood Purif
Systematic Reviews with meta-analysis
Chang’s, 2017, Crit. Care Med
Li’s, 2020, J Crit. Care
Terayama’s, 2017, Surg. Infect
Fujii’s, 2017, Intesiv. Care Med
Cohort Studies based on large calinica database
Iwagami’s, 2016, Blood Purif
Nakamura’s, 2017, Crit. Care
